# The role of atorvastatin on the restenosis process post-PTA in a diabetic rabbit model

**DOI:** 10.1186/s12872-016-0324-1

**Published:** 2016-07-16

**Authors:** Xiaojun Zhou, Yaru Mou, Xue Shen, Tianshu Yang, Ju Liu, Fupeng Liu, Jianjun Dong, Lin Liao

**Affiliations:** Department of Endocrinology, Shandong Provincial Qianfoshan Hospital, Shandong University, No.16766, Jingshi Road, Lixia District, Jinan, 250000 Shandong Province China; Department of Cardiology, Shandong Provincial Hospital affiliated to Shandong University, Shandong University, Jinan, Shandong China; Department of Endocrinology, Shandong Provincial Qianfoshan Hospital, Shandong University of Traditional Chinese Medicine, Jinan, China; Laboratory of Microvascular Medicine, Medical Research Center, Shandong Provincial Qianfoshan Hospital, Shandong University, Jinan, Shandong China; Department of Endocrinology, Qilu Hospital of Shandong University, No.44, wenhuan Road, Lixia District, Jinan, 250000 Shandong Province China

**Keywords:** Atorvastatin, Restenosis, Percutaneous transluminal angioplasty, Peripheral vascular disease, Diabetes

## Abstract

**Background:**

Restenosis remains to be a major limitation of percutaneous transluminal angioplasty (PTA) for diabetic patients with peripheral vascular disease (PVD). Despite of stations routine implements to prevent such progress, its exact effect is unclear.

**Methods and results:**

In our study, balloon was successfully implanted in the iliac artery of atherosclerotic rabbit. Patency of the narrowed artery was interrogated using ultrasound. Atorvastatin or vehicle was administered orally to rabbits from day 0 to day 28 after double-injury surgery. On day 7, day 14, and day 28, restenotic arteries were harvested and processed for histopathlogical analysis. Our data show that, after double-injury surgery, the intima was composed mostly by SMCs at all time course in rabbits undergoing surgery process. Significant increases in stenosis rates were noted from day 7 to day 14 (from 21 ± 5.85 % to 60.93 ± 12.46 %). On day 28 after double-injury surgery, severe restenosis was observed and daily administration of atorvastatin cannot prevent restenosis’ formation (88.69 ± 3.71 % vs. 90.02 ± 3.11 %, *P* > 0.05). The PCNA index and SMCs proliferation were correlated with the scores of the vascular pathology.

**Conclusions:**

Our results indicate that double-injury model can mimic clinical restenosis, based on this model, atorvastatin showed no therapeutic effect on restenosis process in diabetic rabbits after PTA.

## Background

Percutaneous transluminal angioplasty (PTA), despite its widespread use and high initial success rate in diabetes mellitus with peripheral vascular disease (PVD) [1], restenosis, which occurs in up to 70 % patients within one year [2] becomes the limitation for this clinical application. Attempting to pharmacological prevent or reduce it by using antiplatelet agents, corticosteroids, and calcium channel blockers have been unfavorable [3].

Lack of practical restenosis model has limited the possibility to investigate potential therapies. Mounting angioplasty-stenosis animal models have been employed to investigate the pharmacological and mechanical approaches. However, in most studies [4, 5], overstretching angioplasty balloons on normal arteries were developed to reproduce aspects of the human vascular response to PTA (single-injury model) which is applied to build atherosclerosis models, not restenosis models. Therefore, it is crucial to set up a reliable animal model to obtain the ability of investigating the potential therapies to prevent restenosis after PVD patients undergoing PTA.

Statins, a class of 3-hydroxy-3-methylglutaryl (HMG)-coenzyme A (CoA) reductase inhibitors, have both anti-inflammatory and anti-proliferative properties irrespective of their cholesterol-lowering effects [6]. Data from previous studies showed that statins reduced both inflammatory responses [7] and neointimal hyperplasia of arteries in balloon injury animal models (single-injury animal models) [8]. For these reasons, it is clinically used as a routine treatment for PVD patients undergoing PTA to inhibit the process of restenosis. However, in our daily work, we found the clinical efficacy of statins for the restenosis inhibition was minor.

In the present study, we established a double-injury restenosis model in New Zealand white rabbits that mimic the proliferative process of human iliac artery restenosis after PTA with hyperglycemia. Atorvastatin is the most widely prescribed statin drug [9]. Thus, in this study, atorvastatin was used to test how much the administration of statins could contribute to the inhibition of restenosis. By using this animal model, we aimed to investigate the potential therapies of restenosis after PTA in diabetes patients with PVD, or to show insight into the mechanisms of the restenosis process itself.

## Methods

### Animals

This experiment was approved by the Chinese National Institutes of Health, and the protocol was approved by the ethical committee of Qianfoshan Hospital Affiliated to Shandong University. Male New Zealand white rabbits (1.7 kg) were obtained from the Animal Center of Shandong Agriculture Science Academy, China. The rabbits were singly housed in standard rabbit caging and maintained on a 12:12-h light: dark cycle. Atherogenic diet (1 % cholesterol) was given to rabbits 1 week before the experimentation until sacrificed. All rabbits had free access to water throughout the duration of the study.

### Experimental protocol

Induction of experimental diabetes mellitus: after an overnight fasting, 80 mg/kg of freshly prepared alloxan solution was injected intravenously from the marginal ear vein of rabbits [10]. Animals had free access to food and water after the alloxan injection, and an oral solution of 20 % glucose was provided after confirmation of hypoglycemia to prevent hypoglycemic shock. Blood glucose concentrations were measured one week after alloxan injection, animals with blood glucose levels >300 mg/dl were considered as diabetic and enrolled in the experiment.

**Fig. 1:**
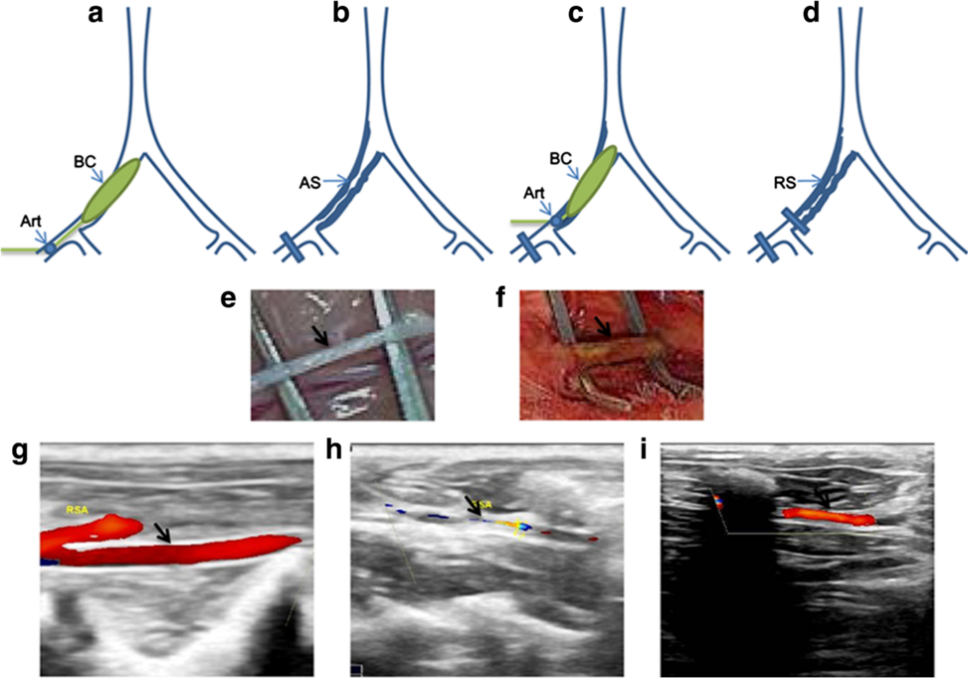
**a** The distal saphenous artery was ligated and a balloon catheter (BC) was advanced into the femoral and iliac arteries through an arteriotomy (Art) in the proximal saphenous artery. **b** Atherosclerotic plaque (AS) was established in the femoral and iliac arteries. **c** After the first surgery, a midsagittal incision was made in the dissected distal ends of the femoral artery. A BC was inserted into the narrowed artery and inflated at the site of stenosis. **d** After the double-injury surgery, restenotic plaque (RS) was established in the post-PTA artery. **e** and **f** Bright field view of control and restenosis arteries, respectively. **g** Color Doppler demonstrated blood flow through non-injured arteries. **h** Color Doppler demonstrated the atherosclerotic plaque’s formation in iliac arteries after the first balloon injury. **i** Color Doppler demonstrated the patency of the stenosed iliac arteries after PTA surgery

Double-injury surgical procedure: (1) Balloon-induced endothelial injury surgery was performed to induce atherosclerotic plaque. One week after diabetes induction, rabbits were anesthetized with phenobarbital. An arteriotomy in the right saphenous artery was made with standard surgical techniques, then a 2.5-mm, wire-guided balloon catheter was inserted and the iliac artery injury was made as previously described [10] (Fig. 1a). The iliac artery developed severe atherosclerotic damage at about 4 weeks after the first surgical procedure, confirmed by ultrasound examination (Visualsonics, Toronto, Canada) (Fig. 1b). (2) PTA was then performed to induce restenotic plaque. Balloon catheter was inserted into the previously balloon-injured iliac artery through the dissected distal ends of the right femoral artery, and PTA was performed at the first injury site (Fig. 1c). Following the above treatment, another color Doppler was developed to demonstrate the patency of the atherosclerotic iliac arteries (Fig. 1i).

Restenosis models successfully induced by double-injury surgery were divided into four groups: 7-day restenosis group (group 1, *n* = 6); 14-day restenosis group (group 2, *n* = 6); 28-day restenosis group (group 3, *n* = 6) and 28-day atorvastatin group (group A, *n* = 6). The atorvastatin dosages (2.5 mg/kg/d)) were determined by previous studies [11, 12]. Atorvastatin therapy started on the day of double-injury surgery and was administered orally by gastric gavage. Six sham-operated diabetic rabbits underwent the same surgical procedure, except that the balloon was not inserted and were applied to the control group (group C).

### Tissue harvest and morphometric analysis

At each time points (7, 14, and 28 days after double-injury surgery), animals were sacrificed (animals in group A and group C were sacrificed on day 28). Blood samples were collected from the ear vain and the levels of serum total cholesterol, triglycerides, low-density lipoprotein, and high-density lipoprotein cholesterol were detected. The injured iliac artery segments were fixed in in 4 % formaldehyde, embedded in paraffin. 5 μm sections were stained with hematoxylin-eosin (HE) for general appearance, Masson’s trichrome for collagen and smooth muscle cells (SMCs), and elastic van-Gieson (EVG) dye for elastin, then observed under microscopy (OLYMPUS FSX100). Morphometric analysis of the intimal growth was performed by the image analysis software Image Pro-Plus Software (Image Pro-Plus 6.0, Media Cybernetics, Silver Spring, MD, USA). Intimal growth was estimated by the ratio of the intimal area to the area bounded by the internal elastic lamina (luminal cross-sectional area narrowing) and the ratio of intimal to medial areas [13, 14].

### Immunohistochemical analysis

Paraffin embedded sections were deparaffinised with Histoclear and rehydrated through graded ethanols. Heat induced antigen retrieval was performed in a microwave oven using Tris-EDTA buffer (10 mM Tris, 1 mM EDTA, 0.05 % Tween 20, pH 9.0) for 20 min and the slides were allowed to cool down prior to blocking with 3 % H_2_O_2_. After blocking with 5 % (v/v) goat serum in phosphate buffered saline (PBS), sections were incubated overnight at 4 °C with the primary antibodies. After a PBS wash, the sections were incubated with secondary antibody at 37 °C for 30 min. Visualization of a positive reaction was by means of a peroxidase substrate solution containing 0.02 % (wt/Vol) H_2_O_2_ and 0.1 % (wt/Vol) 3,3′-diaminobenzidine tetrahydrochloride (ZSBIO, Beijing, China) in PBS to produce a brown color, then the sections were counterstained with hematoxylin. A negative control, with the primary antibody replaced by mouse IgG antibody, was always included. The primary antibodies used were anti-proliferating cell nuclear antigen antibody (PCNA) (Merck, Millipore, Darmstadt, Germany) diluted 1:400 to identify cell proliferation. PCNA positive cells were counted in 10 randomly selected × 200 high-power fields under a microscope (OLYMPUS FSX100). The PCNA index was calculated according to the following formula: number of PCNA positive cells/total cell count × 100 % [15].

### Immunofluorescent staining

Immunofluorescent staining was performed. Briefly, endogenous peroxidase activity was inhibited by incubation with 3 % H_2_O_2_ and blocked with 5 % (v/v) goat serum for 1 h. After washing with PBS, slides were incubated with FITC conjugated-smooth muscle actin antibody (αSMA) (1:100; Abcam, Cambridge, United Kingdom) for four hours. A second wash was followed by preparation of slides in DAPI-Fluoromount-G. Specimens were examined with a fluorescence microscope (OLYMPUS FSX100).

### Statistical analysis

All data are presented as mean ± standard Error (SE). Comparisons were made using one-way ANOVA with SPSS Software (Version 20.0, SPSS China, Shanghai, China). A value of *P* < 0.05 was considered statistically significant.

## Results

### Animals

At the beginning, 45 New Zealand white rabbits were enrolled. After injected with alloxan, 30 generated hyperglycemia successfully, five died and ten were excluded due to inadequate glucose levels. Of the 30 established diabetic rabbits, 24 received double-injury surgery, and all achieved restenosis.

The body weights and blood glucose levels of the diabetic rabbits were measured. Blood glucose concentrations were maintained at levels higher than 300 mg/dl throughout the study. On day 28, the serum total cholesterol level was 18.75 ± 0.44 mmol/l in group C, 18.47 ± 0.54 mmol/l in group 3, and 9.44 ± 0.27 mmol/l in group A. There was no significant difference in the serum total cholesterol level between the group C and group 3, a significant decrease was found between group A and group 3 (*P* < 0.05). Similar findings were observed for the serum LDL-cholesterol level (10.2 ± 0.35 mmol/l in group C, 10 ± 0.34 mmol/l in group 3, and 4.60 ± 0.18 mmol/l in group A), and the serum triglycerides level (2.01 ± 0.04 mmol/l in group C, 2.15 ± 0.05 mmol/l in group 3, and 1.36 ± 0.05 mmol/l in group A). No significant difference of serum HDL-cholesterol levels were found among these groups (0.86 ± 0.02 mmol/l in group C, 0.81 ± 0.03 mmol/l in group 3, and 0.88 ± 0.02 mmol/l in group A). Details were shown in Table 1.

**Table 1:** Animal characteristics

New Zealand	Group C	Group 1	Group 2	Group 3	Group A
White rabbits	(*n* = 6)	(*n* = 6)	(*n* = 6)	(*n* = 6)	(*n* = 6)
Weight (kg)	2.16 ± 0.27	2.04 ± 0.19	2.17 ± 0.31	2.25 ± 0.22	2.30 ± 0.33
Glucose (mmol/l)	19.71 ± 0.92	20.18 ± 0.93	19.95 ± 1.02	19.65 ± 1.18	19.73 ± 0.87
Total cholesterol (mmol/l)	18.75 ± 0.44	18.61 ± 0.48	18.68 ± 0.38	18.47 ± 0.54	9.44 ± 0.27*
LDL-cholesterol (mmol/l)	10.2 ± 0.35	10.3 ± 0.30	10.2 ± 0.48	10 ± 0.34	4.60 ± 0.18*
Serum triglycerides (mmol/l)	2.01 ± 0.04	2.08 ± 0.04	2.05 ± 0.04	2.15 ± 0.05	1.36 ± 0.05*
HDL-cholesterol (mmol/l)	0.86 ± 0.02	0.80 ± 0.02	0.81 ± 0.02	0.81 ± 0.03	0.88 ± 0.02

*P < 0.05 vs Group 3

### Histopathological study and time course of the double-injury model

**Fig. 2:**
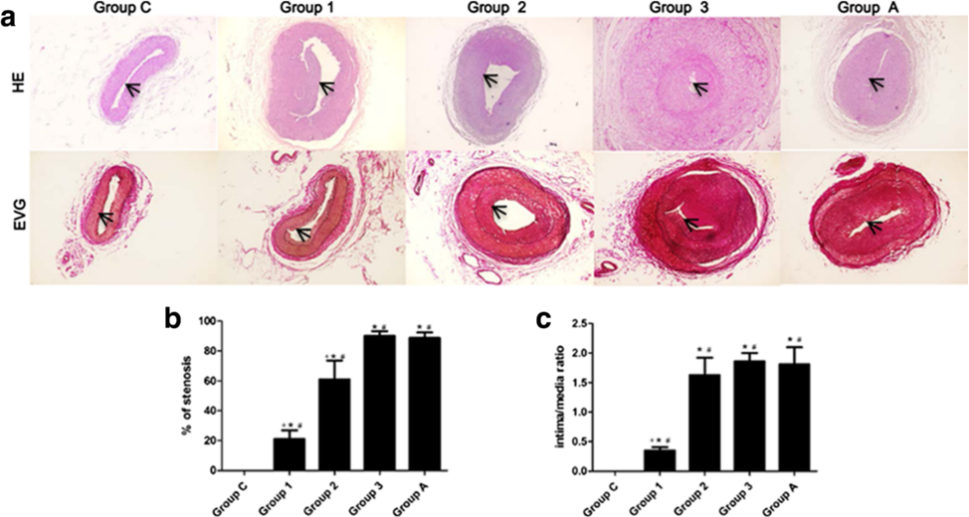
Restenosis’s formation in iliac arteries after double-injury surgery. HE and VGF-stained sections show increasing neointimal formation post double-injury surgery at 7, 14 and 28 days compared to sham-operated controls (*Group C*). Atorvastatin’s inhibitory effect on restenosis was examed as well (*Group A*). **a** Restenosis was measured by the total size of the artery. **b** The stenosis rate. **c** The inimal-to-media area ratio. * *P* < 0.05 vs. group N; # *P* < 0.05 vs. 7-day; + *P* < 0.05 vs. 28-day

The intimal thickening that formed after the primary injury contained SMCs embedded in matrix and served as the substrate for the PTA (Fig. 2a). After the double-injury surgery, there was obvious evidence of trauma, including extensive breaks in the internal elastic laminae and intimal dissections extending into the media.

In the protocol on the time-course of restenosis formation, we used 18 rabbits (6 each for 3 time points after double-injury surgery) to observe the restenosis process. Figure 2a shows that there was a progressive increase in the intima of the iliac arteries of rabbits that underwent double-injury surgery, compared to the iliac arteries of rabbits in group C (*P* < 0.05). The stenosis rate increased from 21 ± 5.85 % to 60.93 ± 12.46 % and 90.02 ± 3.11 % at day 7, 14, 28, respectively (Fig. 2b). Similarly, intima/media ratio increased from 0.35 ± 0.05 to 1.63 ± 0.29 at day 14 and 1.86 ± 0.14 at day 28 (Fig. 2c).

**Fig. 3:**
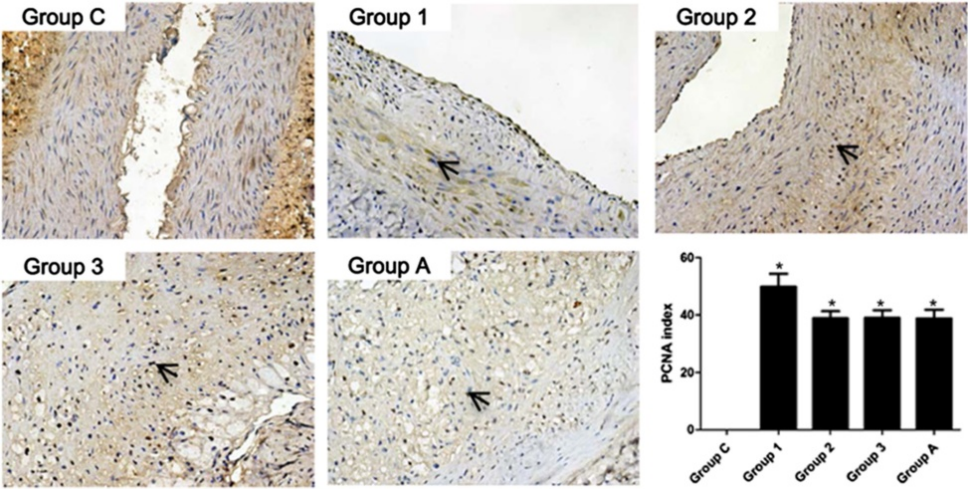
PCNA expression in the double-injury iliac arteries. Magnification 200×. Quantification of positive immunostaining expressed as a ratio of positive/total cells ± standard Error. * *P* < 0.05 vs. group N; # *P* < 0.05 vs. 7-day; + *P* < 0.05 vs. 28-day

**Fig. 4:**
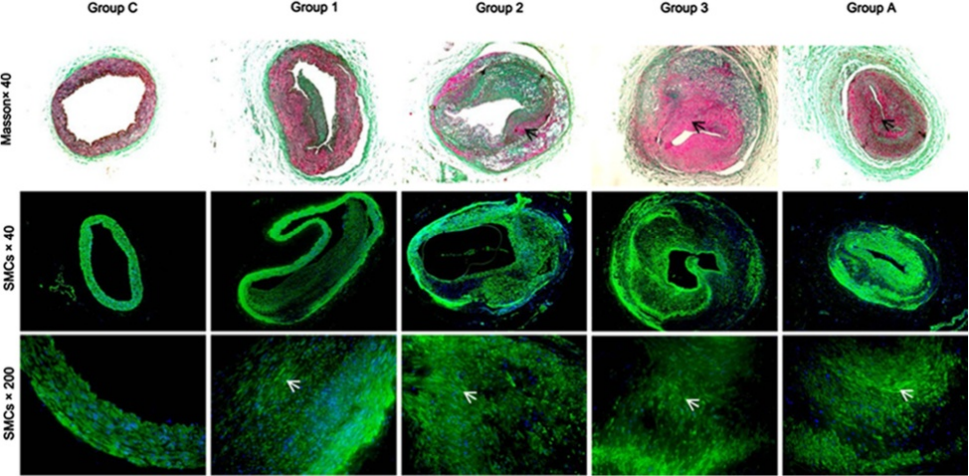
Masson and αSMA immunofluorescence stainings for SMCs in the double-injury iliac arteries. Representative sections are shown. Magnification 40× and 200×

Expression of PCNA was maximally induced at seven days (49.88 ± 4.55 %), then decreased to 38.98 ± 2.32 % on day 14 and stay still on day 28 (Fig. 3). By immunohistochemistry of alpha-actin (a marker for SMCs), it showed that at each time point, the intima was almost entirely contained by SMCs (Fig. 4).

### Effects of atorvastatin on stenosis rate and the intima/media areas in the double-injury model

Twenty eight days atorvastatin treatment in group A, didn’t reduce the increased stenosis rate of the injured iliac arteries of rabbits that underwent double-injury surgery (*P* > 0.05, Fig. 2b). In keeping with the stenosis rate results, the calculated intima/media ratio was significantly increased in group 3, but atrovasatin treatment didn’t reduce the increased intima/media ratio in group A (Fig. 2c).

### Effect of atorvastatin on cell proliferation and SMCs migration

To further elucidate atorvastatin’ effect acts on restenosis in the double-injury model, we performed immunostainings for PCNA for the conditions of cell proliferation and Masson as well as αSMA immunofluorescence stainings for SMCs migration in the neointima. The PCNA index in the injured iliac artery of rabbit in group 3 was significantly increased (*P* < 0.05, Fig. 3). After atorvastatin treatment, the PCNA index remained high and unchanged compared to group A (*P* > 0.05, Fig. 3). As revealed by alpha-actin immunohistochemistry, in samples from both atorvastatin treatment (group A) and non-atorvastatin treatment (group 3) groups, large number of SMCs were observed in the intima, and no significant difference was found between these two groups (*P* > 0.05, Fig. 4).

## Discussion

For many years, efforts to prevent restenosis have been unsuccessful because of the poor understanding of its pathophysiological mechanism [3]. The lack of the ideal restenosis animal model is the major obstacle. Although the single-injury animal model has made important contributions to understanding the mechanisms underlying restenosis [4, 5, 16], it may not reflect the events occurring in atherosclerotic arteries that are treated with balloon angioplasty and subsequently become renarrow.

Histopathologic observation of restenotic tissue from living patients has become readily available with the advent of directional atherectomy [17, 18]. Our double-injury restenosis rabbit model was generated by inducing atherosclerotic plaques in the iliac artery and then deployed PTA on the atherosclerotic rabbits. The histopathology in the restenosis rabbit model mimics human artery restenosis well, including proliferation and migration of SMCs in the restenosis segment which have been identified as the most important histopathological changes in human restenosis, and a similar percentage of proliferating cell in restenotic plaque. Thus, this animal model may provide a pathologic correlation to the human retenosis after PTA treatment and would provide a platform for evaluating anti-restenotic drugs and may carry out basic studies to determine the molecular mechanisms underlying the post-PTA neointimal formation.

The time-course of restenosis was examed in our study. We found that restenotic plaque initiated to form from day 0 to day 7, but developed more rapidly from day 7 to day 14 and this tendency continued to day 28. Previous studies have shown that the migration of SMCs from the tunica media towards the intima which is thought to be a key event in the neointimal formation in restenosis was initiated by balloon injury with a switch of contractile phenotype [19, 20]. This change of phenotype occurred at about one week after the arterial injury [21]. And that may be the reason for the fastest formation of restenotic plaque starting at day 7.

What’s interesting is that the data in our study showed atorvastatin treatment had no effect on the inhibition of SMCs proliferation and migration in the injured iliac artery of resenosis rabbit models, therefore cannot prevent neointimal formation after PTA. This is consistent with our clinical observation.

In fact, some better-controlled clinical trials have made a point that statins lacked therapeutic efficacy on restenosis inhibition. And as the most widely prescribed statin drugs [22–25], atorvastatin cannot arrest the development of restenosis in our study, despite it also has effect on the inhibition of SMCs’ proliferation and migration in vitro [26, 27]. There could be several explanations for the discrepancies of atorvastatin’s effect on restenosis between in vitro and in vivo results. Firstly, experimental exposure of isolated SMCs to atorvastatin in the culture medium may be quite different from in vivo situation, especially that the proliferation and migration of SMCs after PTA is initiated from depth within the arterial media. Therefore, it is possible that the target atorvastatin’s pinpoint has little contribution to the restenosis progress in vivo. Secondly, in vivo, conditions are more complex than those in vitro, different factors and signal pathways may be involved in the pathogenesis of restenosis. Atorvastatin may inhibit some factors or pathways relevant with restenosis inhibition [26, 27], but it is quite possible that other restenosis promoting factors or pathways counterbalance atorvastatin’s effect. Then the net result is atorvastatin cannot significantly inhibit the development of restenosis in vivo. Thirdly, phenotype switch of SMCs results in SMCs’ proliferation and migration cannot be inhibited by atorvastatin.

To our knowledge, this is the first report of the animal restenosis model by using double-injure surgery and investigation of the effect of atorvastatin. This model may support further investigation of the pathophysiological mechanisms underlying restenosis after PTA and research for seeking effective therapeutic interventions.

This study reports preliminary data. Our investigation did not evaluate the fate of the restenosis after PTA at time points greater than 28 days as we were interested in the rapid mimicking ability of restenosis after PTA in animal models. Since the timeline for restenosis of our animal model was significantly shorter than that of human restenosis after PTA, this model had less collaterals.

## Conclusion

In conclusion, we could reproducibly develop restenosis in animal peripheral arteries that mimic human restenosis after PTA. This model may be beneficial for elucidating restenosis mechanisms and seeking for therapeutic interventions. Atorvastatin was demonstrated to have no therapeutic effect on the inhibition of restenosis process. Further studies should be carried out to search for more efficient therapies.

## Abbreviations

CoA, coenzyme A; EVG, van-Gieson; HE, hematoxylin-eosin; HMG, 3-hydroxy-3-methylglutaryl; PCNA, proliferating cell nuclear antigen; PTA, percutaneous transluminal angioplasty; PVD, peripheral vascular disease; SMCs, smooth muscle cells

## References

[1] Apelqvist J, Elgzyri T, Larsson J, Londahl M, Nyberg P, Thorne J (2007). Factors related to outcome of neuroischemic/ischemic foot ulcer in diabetic patients. J Vasc Surg.

[2] Liistro F, Porto I, Angioli P, Grotti S, Ricci L, Ducci K (2013). Drug-eluting balloon in peripheral intervention for below the knee angioplasty evaluation (DEBATE-BTK): a randomized trial in diabetic patients with critical limb ischemia. Circulation.

[3] Forte A, Rinaldi B, Berrino L, Rossi F, Galderisi U, Cipollaro M (2014). Novel potential targets for prevention of arterial restenosis: insights from the pre-clinical research. Clin Sci (Lond).

[4] Brito LA, Chandrasekhar S, Little SR, Amiji MM (2010). Non-viral eNOS gene delivery and transfection with stents for the treatment of restenosis. Biomed Eng Online.

[5] Santulli G, Wronska A, Uryu K, Diacovo TG, Gao M, Marx SO (2014). A selective microRNA-based strategy inhibits restenosis while preserving endothelial function. J Clin Invest.

[6] Menge T, Hartung HP, Stuve O (2005). Statins--a cure-all for the brain?. Nat Rev Neurosci.

[7] Walter DH, Schachinger V, Elsner M, Mach S, Auch-Schwelk W, Zeiher AM (2000). Effect of statin therapy on restenosis after coronary stent implantation. Am J Cardiol.

[8] Gellman J, Ezekowitz MD, Sarembock IJ, Azrin MA, Nochomowitz LE, Lerner E (1991). Effect of lovastatin on intimal hyperplasia after balloon angioplasty: a study in an atherosclerotic hypercholesterolemic rabbit. J Am Coll Cardiol.

[9] Kawahara T, Nishikawa M, Kawahara C, Inazu T, Sakai K, Suzuki G (2013). Atorvastatin, etidronate, or both in patients at high risk for atherosclerotic aortic plaques: a randomized, controlled trial. Circulation.

[10] Zhou X, Dong J, Zhang L, Liu J, Dong X, Yang Q (2015). Hyperglycemia has no effect on restenosis development after PTA in diabetic rabbit model. J Endocrinol.

[11] Mangaloglu L, Cheung RC, Van Iderstine SC, Taghibiglou C, Pontrelli L, Adeli K (2002). Treatment with atorvastatin ameliorates hepatic very-low-density lipoprotein overproduction in an animal model of insulin resistance, the fructose-fed Syrian golden hamster: evidence that reduced hypertriglyceridemia is accompanied by improved hepatic insulin sensitivity. Metabolism.

[12] Wong V, Stavar L, Szeto L, Uffelman K, Wang CH, Fantus IG (2006). Atorvastatin induces insulin sensitization in Zucker lean and fatty rats. Atherosclerosis.

[13] Rodriguez-Menocal L, Wei Y, Pham SM, St-Pierre M, Li S, Webster K (2010). A novel mouse model of in-stent restenosis. Atherosclerosis.

[14] Tominaga R, Kambic HE, Emoto H, Harasaki H, Sutton C, Hollman J (1992). Effects of design geometry of intravascular endoprostheses on stenosis rate in normal rabbits. Am Heart J.

[15] Mohammed A, Janakiram NB, Madka V, Ritchie RL, Brewer M, Biddick L (2014). Eflornithine (DFMO) prevents progression of pancreatic cancer by modulating ornithine decarboxylase signaling. Cancer Prev Res (Phila).

[16] Yin RX, Yang DZ, Wu JZ (2014). Nanoparticle drug- and gene-eluting stents for the prevention and treatment of coronary restenosis. Theranostics.

[17] Edlin RS, Tsai S, Yamanouchi D, Wang C, Liu B, Kent KC (2009). Characterization of primary and restenotic atherosclerotic plaque from the superficial femoral artery: potential role of Smad3 in regulation of SMC proliferation. J Vasc Surg.

[18] Nakano M, Otsuka F, Yahagi K, Sakakura K, Kutys R, Ladich ER (2013). Human autopsy study of drug-eluting stents restenosis: histomorphological predictors and neointimal characteristics. Eur Heart J.

[19] Sampietro ML, Trompet S, Verschuren JJ, Talens RP, Deelen J, Heijmans BT (2011). A genome-wide association study identifies a region at chromosome 12 as a potential susceptibility locus for restenosis after percutaneous coronary intervention. Hum Mol Genet.

[20] Zargham R (2008). Preventing restenosis after angioplasty: a multistage approach. Clin Sci (Lond).

[21] Zargham R, Pepin J, Thibault G (2007). alpha8beta1 Integrin is up-regulated in the neointima concomitant with late luminal loss after balloon injury. Cardiovasc Pathol.

[22] Bertrand ME, McFadden EP, Fruchart JC, Van Belle E, Commeau P, Grollier G (1997). Effect of pravastatin on angiographic restenosis after coronary balloon angioplasty. The PREDICT Trial Investigators. Prevention of restenosis by Elisor after transluminal coronary angioplasty. J Am Coll Cardiol.

[23] Kleemann A, Eckert S, von Eckardstein A, Lepper W, Schernikau U, Gleichmann U (1999). Effects of lovastatin on progression of non-dilated and dilated coronary segments and on restenosis in patients after PTCA. The cholesterol lowering atherosclerosis PTCA trial (CLAPT). Eur Heart J.

[24] Serruys PW, Foley DP, Jackson G, Bonnier H, Macaya C, Vrolix M (1999). A randomized placebo-controlled trial of fluvastatin for prevention of restenosis after successful coronary balloon angioplasty; final results of the fluvastatin angiographic restenosis (FLARE) trial. Eur Heart J.

[25] Weintraub WS, Boccuzzi SJ, Klein JL, Kosinski AS, King SB, Ivanhoe R (1994). Lack of effect of lovastatin on restenosis after coronary angioplasty. Lovastatin Restenosis Trial Study Group. N Engl J Med.

[26] Chandrasekar B, Mummidi S, Mahimainathan L, Patel DN, Bailey SR, Imam SZ (2006). Interleukin-18-induced human coronary artery smooth muscle cell migration is dependent on NF-kappaB- and AP-1-mediated matrix metalloproteinase-9 expression and is inhibited by atorvastatin. J Biol Chem.

[27] Li M, Liu Y, Dutt P, Fanburg BL, Toksoz D (2007). Inhibition of serotonin-induced mitogenesis, migration, and ERK MAPK nuclear translocation in vascular smooth muscle cells by atorvastatin. Am J Physiol Lung Cell Mol Physiol.

